# Supplementary material for: 3-Iodothyronamine Affects Thermogenic Substrates’ Mobilization in Brown Adipocytes
Source: Biology (Basel). 2020 May 4;9(5):95. doi: 10.3390/biology9050095 (PMC7285105; doi:10.3390/biology9050095)
Supplement: Supplementary file 1 [file biology-09-00095-s001.pdf]

biology MDPI

Supplementary Materials

Table 1. *Primary and secondary antibodies used for immunofluorescence (IF) and Western-blot analysis (WB).*

Name product	Dilution	Supplier
Anti-β1AR	1:500 (WB)	Santa Cruz Biotechnology Inc. Dallas, TX, USA (SC-568)
Anti-β3AR	1:500 (WB)	Santa Cruz Biotechnology Inc. Dallas, TX, USA (SC-515763)
Anti-UCP1	1:500 (WB)	Santa Cruz Biotechnology Inc. Dallas, TX, USA (SC-293414)
Anti-p-AKT S473	1:1000 (WB)	Cell Signaling Technology, Denver, CO, USA (#4060)
Anti-AKT	1:1000 (WB)	Cell Signaling Technology, Denver, CO, USA (#9272)
Anti-p-PKAαβγ (Thr 198)	1:1000 (WB)	Santa Cruz Biotechnology Inc. Dallas, TX, USA (SC-32968)
Anti-PKA	1: 1000 (WB)	BD Transduction Laboratories, San Jose, CA) USA (610980)
Anti-p-P38	1:400 (WB)	Cell Signaling Technology, Denver, CO (USA) (#4511)
Anti-P38	1:500 (WB)	Santa Cruz Biotechnology Inc. Dallas, TX, USA (SC-7972)
Anti-p-CREB	1:1000 (WB)	Cell Signaling Technology, Denver, CO, USA (#9162)
Anti-CREB	1:1000 (WB)	

		Cell Signaling Technology, Denver, CO, USA (#9192)
Anti-GLUT4	1:500 (WB)	Santa Cruz Biotechnology Inc. Dallas, TX, USA(SC-7938)
Anti-GLUT1	1:100 (IF)	Santa Cruz Biotechnology Inc. Dallas, TX, USA
Anti AMPK	1:1000 (WB)	Santa Cruz Biotechnology Inc. Dallas, TX, USA (SC-74461)
Anti p- AMPK	1:1000 (WB)	Cell Signaling Technology, Denver CO, USA (#2535S)
Anti-GAPDH	1:7000 (WB)	Merk-Millipore,Darmstadt Germany) (AB2302)
Anti-MouseIgG Alexa Fluor 594	1:150 (IF)	Thermo Fisher scientific, USA

Figure S1. The expression levels of GLUT1

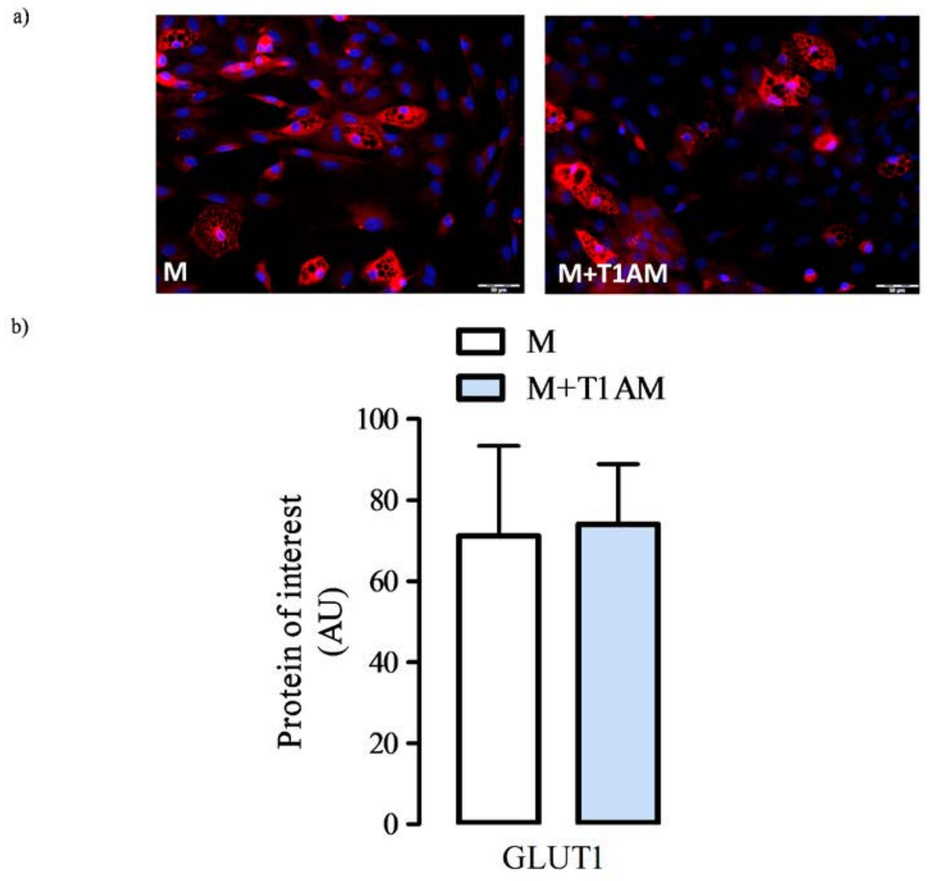

M and M+T1AM cells cultured as described in Methods on glass coverslip were used for the immunofluorescence staining of GLUT1 (**a**) representative experiment is showed; The target protein is depicted in red, cell nuclei are stained in blue (DAPI); image magnification 20X; scale bar 50 µm. (**b**) The densitometric analysis of immunofluorescence determination of GLUT1 is reported as Arbitrary Units of Fluorescence (AU) as described in "Methods".
